# Metabolic Phenotypes of Hypoxic-Ischemic Encephalopathy with Normal vs. Pathologic Magnetic Resonance Imaging Outcomes

**DOI:** 10.3390/metabo10030109

**Published:** 2020-03-14

**Authors:** José David Piñeiro-Ramos, Antonio Núñez-Ramiro, Roberto Llorens-Salvador, Anna Parra-Llorca, Ángel Sánchez-Illana, Guillermo Quintás, Nuria Boronat-González, Juan Martínez-Rodilla, Julia Kuligowski, Máximo Vento, The HYPOTOP Study Group

**Affiliations:** 1 Neonatal Research Group, Health Research Institute Hospital La Fe, Avenida Fernando Abril Martorell 106, 46026 Valencia, Spain; jose_pineiro@iislafe.es (J.D.P.-R.); annaparrallorca@gmail.com (A.P.-L.); asanchezillana@gmail.com (Á.S.-I.); juan.martinez.rodilla@gmail.com (J.M.-R.); maximo.vento@uv.es; 2 Division of Neonatology, University & Polytechnic Hospital La Fe, Avenida Fernando Abril Martorell 106, 46026 Valencia, Spain; aguillermo9nr@hotmail.com (A.N.-R.); nubogon@hotmail.com (N.B.-G.); 3 Division of Radiology and Imaging, University & Polytechnic Hospital La Fe, Avenida Fernando Abril Martorell 106, 46026 Valencia, Spain; llorens_rob@gva.es; 4 Health & Biomedicine Unit, Leitat Technological Center, Parc Cientific Barcelona, 08028 Barcelona, Spain; gquintas@leitat.org; 5 Unidad Analítica, Health Research Institute La Fe, Avenida Fernando Abril Martorell 106, 46026 Valencia, Spain

**Keywords:** liquid chromatography–time-of-flight mass spectrometry (LC-TOFMS), neonatal brain injury, perinatal asphyxia, untargeted metabolomics

## Abstract

Hypoxic-Ischemic Encephalopathy (HIE) is one of the most relevant contributors to neurological disability in term infants. We hypothesized that clinical outcomes of newborns with (HIE) can be associated with changes at plasma metabolic level enabling the detection of brain injury. Plasma samples of a cohort of 55 asphyxiated infants who evolved to moderate/severe HIE were collected between birth and completion of therapeutic hypothermia (TH). Samples were analyzed employing a quantitative gas chromatography–mass spectrometry method for the determination of lactate and pyruvate and an untargeted liquid chromatography–time-of-flight mass spectrometry method for metabolic fingerprinting. Brain injury was assessed employing magnetic resonance imaging (MRI). A critical assessment of the usefulness of lactate, pyruvate, and pyruvate/lactate for outcome prediction was carried out. Besides, metabolic fingerprinting identified a dynamic perturbation of eleven metabolic pathways, including amino acid and purine metabolism, and the steroid hormone biosynthesis, in newborns with pathologic MRI outcomes. Although data suggest the usefulness of lactate and pyruvate monitoring during 72 h for discerning outcomes, only the steroid hormone biosynthesis pathway was significantly altered in early plasma samples (i.e., before the initiation of TH). This study highlights pathways that might potentially be targeted for biomarker discovery or adjuvant therapies to be combined with TH.

## 1. Introduction

Hypoxic-Ischemic Encephalopathy (HIE) is the neurological consequence of impaired blood flow and/or gas exchange during birth [1]. Decreased cerebral perfusion and oxygenation trigger a sequence of acute cerebral metabolic alterations that characterize the primary energy failure phase of HIE. After resuscitation, the process undergoes a latent phase that lasts up to 6 h. This stage is followed by a secondary energy failure phase that enhances cerebral injury and may last for days, weeks, or even months [2].

HIE is one of the most relevant contributors to neurological disability in the pediatric age. In high-income countries, the incidence of HIE is around 1.6 per 1000 infants, while the risk is around 10-fold higher in low-income countries [3]. Therapeutic hypothermia (TH) initiated within 6 h of birth constitutes the standard of care for infants with moderate or severe HIE reducing mortality and improving neurodevelopmental outcome in survivors [4].

At present, no bedside test is available for an accurate and early diagnosis of HIE. In the delivery room, physicians rely on prenatal clinical information and serial Apgar scores with special emphasis on neurological assessment. In addition, cord blood gases, metabolic acidosis, and increased lactacidemia further reflect the severity of anaerobic metabolism [2]. The modified Sarnat staging system grades neonatal HIE according to the severity of the brain insult [5]. Later on, amplitude-integrated electroencephalography (aEEG) and/or multichannel EEG and cranial ultrasound will confirm the diagnosis [6]. Magnetic resonance imaging (MRI), including diffusion weighted imaging and spectroscopy, performed 5–7 days after birth, is the cornerstone for neurodevelopmental outcome prediction [7]. Particularly, time-dependent reductions in brain N-acetyl aspartate and creatinine concentrations at 18–96 h and 7–14 days are able to accurately predict adverse outcomes, whereas elevated cerebral lactate, glutamate, and glutamine concentrations have been found to be transient [8]. However, unstable neonates may not tolerate transport necessary for MRI or MRI scanning duration. Moreover, hypothermia may alter EEG recordings and limit its early predictive capacity [2].

HIE has been associated with damage to the integrity of the blood–brain barrier and, hence, brain injury could be reflected in peripheral blood samples [9]. In the search for blood biomarkers capable of determining the degree of brain injury and providing prognostic information, an array of different molecules has been proposed including neuronal tissue specific proteins, proteins involved in the pathogenesis of traumatic brain injury, excitatory amino acids, lipid peroxidation products, specific microRNAs, and inflammatory markers [2,6]. Despite the potential of these markers, few small pilot studies have validated their utility in the clinical practice [10] and none has been added to clinical guidelines [11].

Blood lactate, a hallmark of anaerobic metabolism, has been traditionally employed as surrogate marker for tissue hypoxia and/or ischemia. Pyruvate dehydrogenase controls the metabolic flux between the tricarboxylic acid cycle and glycolysis. Under hypoxic conditions, glucose is metabolized into lactate and exported to the extracellular compartment [12]. In infants with moderate/severe brain injury, a very strong correlation was found between serum and cerebral lactate concentrations at the basal ganglia, thalamus, and gray matter regions during TH [12]. It has been suggested that monitoring lactate could be useful for assessing the degree of HIE and even predict long-term neurodevelopmental outcomes [13]. However, these speculations were not based on adequately powered clinical studies.

Comprehensive metabolic phenotyping has been increasingly employed for identifying possible therapeutic targets, and the discovery of biomarkers to support treatment selection, patient stratification, and enhanced diagnosis [11]. However, metabolomics has only been scarcely used for progressing our understanding of the multilayer effects of HIE and TH on the newborn [14]. ^1^H-nuclear magnetic resonance was used to characterize umbilical cord blood samples from infants with perinatal asphyxia or HIE compared to matched healthy controls, revealing significant changes in 18 metabolites [15]. A panel of four metabolites showed potential for predicting HIE severity according to Sarnat staging; however, it was not found to be superior to EEG or the Sarnat score for predicting neurodevelopmental outcome at three years of age [16]. Umbilical cord blood of a similar cohort of newborns with signs of perinatal asphyxia and HIE as well as matched healthy controls was studied employing direct infusion Fourier-transform ion cyclotron resonance mass spectrometry (MS) [17]. Eight putatively annotated metabolites were identified to be significantly altered in newborns with HIE and four metabolites differentiated across severity grades of HIE based on continuous multichannel EEG monitoring at 24 h of life. In a longitudinal study involving six infants with mild/severe HIE, a specific metabolic profile was identified in urine samples as compared to healthy control subjects on Days 1 and 3 after birth [18].

The aim of this study was to elucidate dynamic metabolic changes in a cohort of newborn infants evolving to moderate/severe HIE undergoing TH in which hypoxia–ischemia-induced brain injury was assessed employing MRI. This study describes for the first time the evolution of the neonatal plasma metabolome of HIE covering the time period between birth and completion of TH treatment. The objectives of the present study were focused on preselecting disease-specific pathways at the phenotypic level that might be suitable for enabling an identification of those infants at risk of developing brain injury within the infants that qualify for hypothermia treatment as well as for monitoring the individual response to treatment and elucidating new potential therapeutic targets.

## 2. Results

### 2.1. Patient Characteristics

Population characteristics are shown in Table 1. There were only minor, non-significant differences between the control and the pathologic group, i.e., a higher proportion of males in the control group (59% vs. 42%) and higher proportion of C-section in the pathologic group (64% vs. 41%). Regarding the Sarnat staging, in the normal group, 86% of infants were initially identified as Sarnat 2, whereas, in the pathologic group, the proportion was 58% (*p*-value = 0.05). Although the analysis of the effect of topiramate vs. placebo was out of the scope of this study, the inclusion of a similar proportion of individuals from both treatment groups (*p*-value = 0.7) allowed compensating for the impact (confounder) of potential effects of pharmacological treatment on the metabolome.

### 2.2. Plasma Lactate and Pyruvate Levels

**Figure 1:**
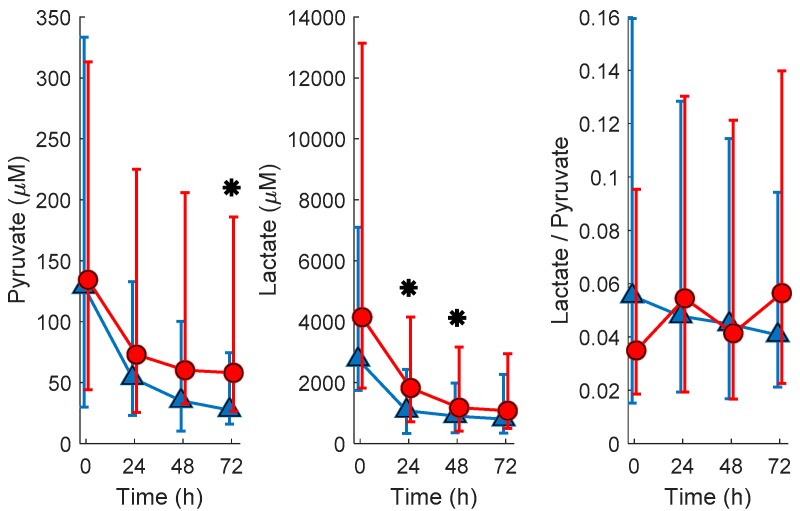
Evolution of pyruvate and lactate concentrations and lactate/pyruvate in plasma samples from newborns with normal (blue ∆ and error bars are median and 25th and 75th percentile, respectively) and pathologic (red o and error bars are median and 25th and 75th percentile, respectively) MRI outcomes. Note: normal vs. pathologic at 0 h *N* = 13 and 23; at 24 h *N* = 20 and 30; at 48 h *N* = 22 and 28; and at 72 h *N* = 20 and 32, respectively; * indicates *p*-vales below 0.05 (Wilcoxon rank sum test) for comparing normal vs. pathologic MRI outcomes; blood samples were extracted at birth and at 24, 48, and 72 h after the administration of the first dose of medication/placebo.

The evolution of lactate and pyruvate plasma concentrations as well as lactate/pyruvate with time are shown in Figure 1. For lactate, as well as pyruvate, median concentrations at all studied time points were higher in newborns with pathologic MRI outcomes. To assess the potential of lactate, pyruvate, and lactate/pyruvate levels determined in blood before the initiation of TH and 24, 48, and 72 h after initiation of TH for predicting pathologic MRI outcomes, groups were compared employing the Wilcoxon rank sum test. Newborns with pathologic MRIs had significantly higher levels of lactate at 24 and 48 h as well as pyruvate at 72 h. Lactate/pyruvate did not show a clear trend with time and was not useful for discriminating between normal and pathologic MRI outcomes at any studied time point.

### 2.3. Untargeted Metabolomic Analysis

**Figure 2:**
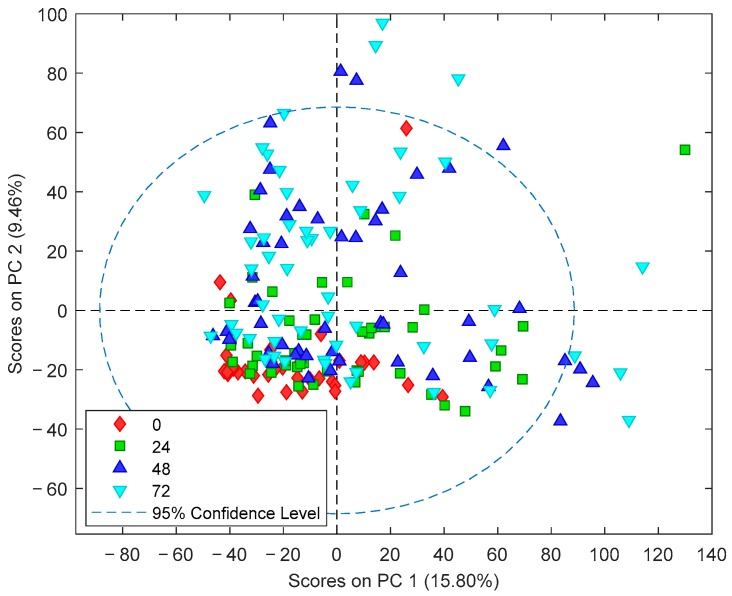
PCA scores plot of plasma metabolomic fingerprints. Note: Blood samples were extracted at birth and at 24, 48, and 72 h after the administration of the first dose of medication/placebo.

Figure 2 shows the Principal Component Analysis (PCA) scores plot of plasma metabolomic analysis collected at the different time points. The strong impact of time on the plasma metabolome can be observed in direction of principal component (PC) 2 (9.5% of the total variance). Based on this observation, further data analysis was stratified by the sampling time point.

**Figure 3:**
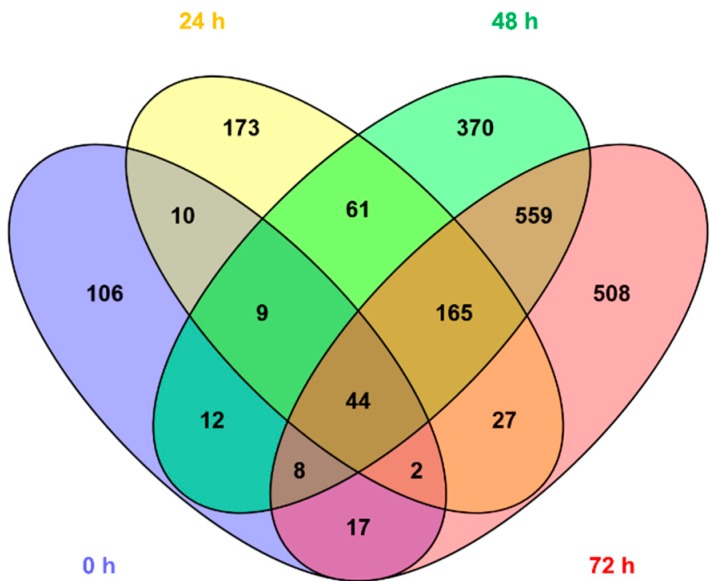
Venn diagram showing the overlap among altered metabolic features in plasma samples collected 0, 24, 48, and 72 h. Note: Blood samples were extracted at birth and at 24, 48, and 72 h after the administration of the first dose of medication/placebo.

**Figure 4:**
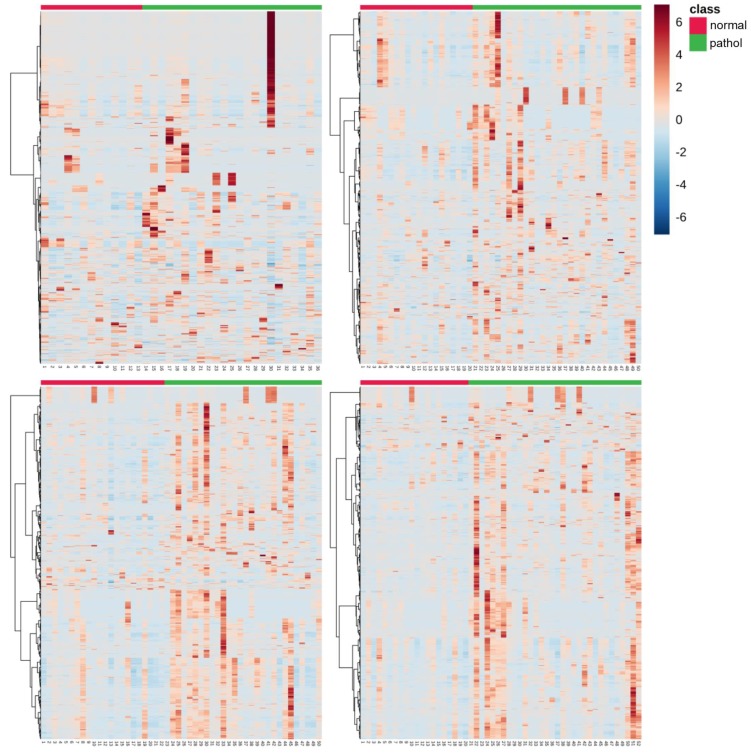
Heat maps of altered plasma metabolic features (*N* = 2071) in samples collected at: 0 h (top, left); 24 h (top, right); 48 h (bottom, left); and 72 h (bottom, right). Note: normal vs. pathologic at 0 h *N* = 13 and 23; at 24 h *N* = 20 and 30; at 48 h *N* = 22 and 28; and at 72 h *N* = 20 and 32, respectively; blood samples were extracted at birth and at 24, 48, and 72 h after the administration of the first dose of medication/placebo.

Figure 3 shows the number of features with different mean values (*t*-test, *α* = 0.05) comparing normal vs. pathologic outcomes as determined by MRI. It can be observed that the number of altered features increases with time. Whereas 208 (2.6%) of the total of 8122 detected features showed different mean concentrations in blood before the initiation of TH, 24 h after the initiation of TH, already 491 (6.0%) were altered; 48 h and 72 h after the initiation of TH, this value further increased to 1228 (15.1%) and 1330 (16.4%), respectively. Furthermore, the pattern of affected metabolites shifted over time with only 65 of the altered features shared between blood before the initiation of TH and 24 h samples, 279 between 24 and 48 h samples, and 776 between 48 and 72 h samples. This indicates that the alteration of the metabolome in newborns with pathologic MRI outcomes rapidly changes at early time points and slowly stabilizes thereafter. In Figure 4, heat maps of the intensities of 2071 metabolic features that showed different distributions between normal and pathologic groups in at least one of the sampling times are depicted. Visual inspection of heat maps confirms an increase of HIE specific plasma metabolite patterns with time: whereas no clear patterns are observed from metabolites detected in blood before the initiation of TH, differences are visible after 24 h and become more evident after 48 and 72 h.

**Figure 5:**
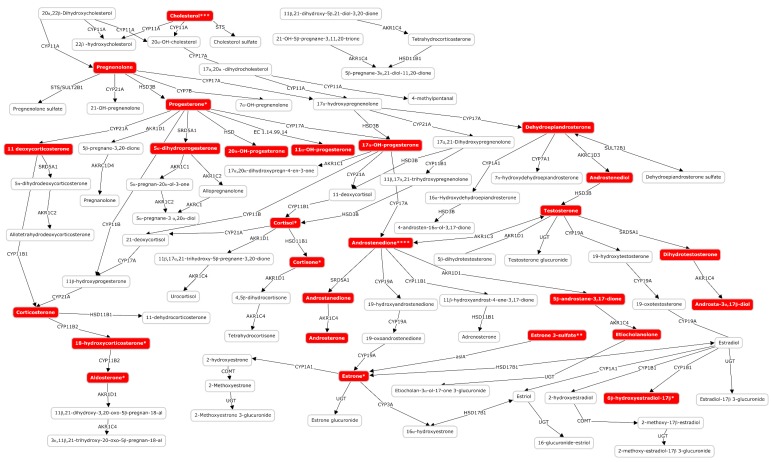
Altered metabolites of the steroid hormone biosynthesis pathway in plasma samples from newborns suffering from HIE at birth and 24, 48, and 72 h after the administration of the first dose of medication/placebo. Note: * altered only at 24, 48, and 72 h; ** altered only at 48 and 72 h; *** altered only at 48 h; **** altered only at 72 h.

Table 2 summarizes the metabolic pathways significantly altered in at least one of the studied time points and Table S1 shows the annotation of altered metabolites detected in plasma samples from newborns with HIE and normal vs. pathologic MRI outcomes. In line with previous observations, different pathways were affected in blood before the initiation of TH as compared to the remaining sampling times. Again, the number of altered pathways varies with time with three, two, five, and nine altered pathways in the first blood sample collected, and 24, 48, and 72 h after the initiation of TH, respectively. In blood collected before the initiation of TH, purine, seleno amino acid metabolism, and steroid hormone synthesis were affected in newborns with pathologic MRI outcomes. Later, additional pathways mainly related to amino acid metabolism were altered. Only one metabolic pathway, i.e., steroid hormone biosynthesis, remained altered during the whole study period. Figure 5 shows the steroid hormone synthesis pathway and its intermediates highlighting metabolites detected and/or altered in this study at the four studied time points.

## 3. Discussion

The HYPOTOP trial was designed to minimize shortcomings of single center trials, thus improving the generalizability of the observations. This is underpinned by the homogeneity of the clinical and demographic parameters between both study groups (normal vs. pathologic MRI outcome), as shown in Table 1. For the quantitative determination of lactate and pyruvate, a targeted gas chromatography–mass spectrometry (GC-MS) method was used, whereas untargeted fingerprinting was carried out using LC-TOFMS. For lactate and pyruvate concentrations in plasma samples collected before the initiation of TH, poor predictive values were found, whereas, during TH, lactate (24 and 48 h) and pyruvate (72 h) were able to discriminate between newborns with confirmed brain injury and normal outcomes, as shown in Figure 1. Optimum cut-off values from receiver operating characteristic (ROC) curves of 1.1 mM and 51.4 μM for lactate at 24 and 48 as well as pyruvate at 72 h, respectively, were obtained for the discrimination between both groups. The difference in concentration levels of lactate between newborns with normal and pathologic MRI outcomes decreased over time, whereas an increase was noted for pyruvate concentrations. Due to the high capacity for lactate transport into and out of the brain, blood lactate has been employed as surrogate marker for brain tissue hypoxia and/or ischemia. It has been demonstrated that serum lactate strongly correlates with cerebral lactate concentrations at the basal ganglia, thalamus, and gray matter regions measured during TH in newborns with moderate to severe brain injury [12]. In accordance with findings on blood lactate, the relative increase in serum lactate dehydrogenase concentrations between Days 1 and 3 was associated with central gray matter lesions [19]. Moreover, lactate and pyruvate concentrations were found to be elevated in plasma from newborns with HIE undergoing TH at 48 h as compared to a control group of healthy term infants [20] and serum lactate was significantly elevated at 72 h of life in newborns with poor neurodevelopmental outcomes [21]. Lactate levels acquired within the first hour of life in combination with serial measurements of lactate have been proposed as predictors of moderate/severe HIE [22] and prolonged time to normalization of serum lactate occurred in severe HIE with prolonged electrographic seizures [23]. In contrast, to date, no literature is available on blood pyruvate levels in newborn infants with HIE. In experimental studies, both concentrations of cerebral pyruvate and lactate were found to be elevated in hypoxia and hypoxia–ischemia [13].

A complementary analytical approach aimed at exploiting the potentials of untargeted metabolomic fingerprinting for enhancing the current understanding of the complex perturbations of hypoxia–ischemia on the individual, phenotypic level and its impact on the pathophysiology of HIE. The importance of repeated phenotypic monitoring should be highlighted as it is well known that HIE passes through different phases and TH induces a reduction in cerebral and whole-body metabolism [2]. Furthermore, the transition of the neonate from hypoxic in utero conditions to a relatively oxygen-rich environment induces changes in the metabolome. In an animal model, 23% of the detected metabolites showed a significant change in concentration when comparing umbilical cord blood to blood samples drawn until 72 h after birth [24]. In the present study, a clear effect of the time elapsed from birth until sample collection has been observed (see Figure 2), with the pattern of injury becoming more visible as time evolves (see Figure 3; Figure 4). This was further corroborated by the results of pathway analysis shown in Table 2 and Table S1, where an upward trend in the number of altered pathways with time was observed while the pattern of injury emerges during secondary energy failure.

We demonstrated that the early evolving blood metabolome provides useful information for the discrimination of newborns who will show pathologic MRI patterns after TH. In the first blood sample analyzed in this study, collected before initiation of TH, levels of two metabolites of the purine metabolism, i.e., xanthine and its derived nucleoside xanthosine, were found to be altered. Purine metabolism has been described repeatedly to be affected in animal experiments on hypoxia/reoxygenation [25] as well as newborns with HIE [17]. Interestingly, xanthine is an oxygen-dependent downstream product of hypoxanthine, which in turn is a hallmark of hypoxia and has been identified as a free radical generator, playing a key-role in oxidative stress-associated diseases of the newborn [25]. Furthermore, the steroid hormone biosynthesis pathway was affected over the whole study period in newborns with HIE and pathological MRI outcomes, but became especially relevant during TH. In agreement with our observations (see Table S1), a significant increase of neonatal 17-hydroxyprogesterone levels has been reported in blood samples from infants suffering from birth asphyxia and neonatal seizures [26]. Although the influence of drugs administered for sedation, or antibiotics and vasopressor drugs cannot be ruled out, the observed effect might likely be linked to the response to stress and pain that inevitably triggers a hormonal response affecting epinephrine, norepinephrine, and cortisol levels. On the other hand, the use of hormones has been proposed as therapeutic drugs in the context of HIE. In experimental studies, the administration of glucocorticoids [27] and progesterone [28] ameliorated neonatal hypoxic-ischemic brain injury.

At later time points in samples collected during TH, a total of eight pathways involving amino acid metabolism, synthesis, and degradation have been observed to be perturbed when comparing between normal vs. pathologic groups. In the literature, several reports on the perturbation of amino acids in hypoxia and hypoxia–ischemia in experimental models and neonates can be found [14] and the accumulation of excitatory amino acids (glutamate and aspartate) in cerebral tissue during the acute phase as well as secondary energy failure have been extensively described [2]. In umbilical cord blood of newborns with HIE in comparison to a healthy control group, alanine, aspartate, and glutamate metabolism, as well as phenylalanine metabolism, were found to be altered [17] and changes in metabolite levels involved arginine and proline metabolism (i.e., proline [29]), nitrogen metabolism (i.e., phenylalanine [29] and tryptophan [17]), and phenylalanine metabolism (i.e., phenylalanine [29] and succinate [15,16,17]) were observed.

This study has some limitations. Although TH is a routine clinical practice, patients need to be transferred to level III NICUs to receive this treatment. Consequently, umbilical cord blood could not be collected from these patients. This is an intrinsic problem of studies on infants with HIE and the exclusion of outborn infants would dramatically increase the time needed for the recruitment of a representative number of subjects. In addition, this study does not include a healthy control group or newborns with asphyxia or classified as mild HIE (Sarnat 1), as only newborns subjected to hypothermia treatment were included. There is risk of adverse neurodevelopmental outcome of mild HIE newborns, but, up to now, there is no evidence to support cooling in these babies, and therefore we consider that the study of the metabolic fingerprint of those infants in correlation with the clinical outcomes would be of interest [30]. Furthermore, metabolomic coverage could be increased by taking advantage of complementary methods and analytical platforms. The use of complementary methodological approaches might help to achieve a more comprehensive view of the global effects of HIE on the plasma metabolome. Before the findings described in this study can be translated to the clinic, an exhaustive validation of the results is necessary involving the identification and quantification with primary standards following guidelines of regulators such as the U.S. Food & Drug Administration and European Medicines Agency.

We report the first metabolomic study involving human subjects and serial sample collections in HIE for modeling brain injury as confirmed with MRI. Herein, the time-dependent evolution of lactate and pyruvate levels in newborns with HIE undergoing TH is shown. Our data underpin the usefulness of blood lactate with a cut-off value of 1.1 mM between 24 and 48 h and pyruvate with a cut-off value of 51.4 μM at 72 h for anticipating favorable/unfavorable MRI outcomes. Although these findings need to be validated in independent cohorts, these biomarkers could be easily implemented within clinical routine care, as portable devices for point-of-care testing are commercially available. However, at very early sampling time points (before initiation of TH), the measurement of those parameters does not support outcome prediction. Metabolic pathway analysis revealed the time-dependent perturbation of several pathways. Purines (i.e., xanthine and xanthosine) were found to be altered in blood samples from newborns with pathologic MRI outcomes drawn before the initiation of TH and the steroid hormone biosynthesis pathway has been significantly altered during the whole study period comprising early samples collected shortly after birth and until completion of TH after 72 h. Hence, this study provides evidence of the usefulness of metabolites from both pathways as candidate biomarkers to be evaluated in future clinical validation studies as they might allow an early prediction of central nervous system impairment reflected in an anatomic alteration detectable by MRI. In depth knowledge of metabolic alterations might furthermore support studies targeting the development of adjuvant therapies to be combined with TH, e.g., the administration of allopurinol and steroid hormones.

## 4. Materials and Methods

### 4.1. Study Approval

The study was approved by the Ethics Committee for Biomedical Research of the Health Research Institute La Fe (Valencia, Spain) and registered as EudraCT 2011-005696-17 under the abbreviation HYPOTOP. Informed consent was obtained from parents of all participants and all methods were performed in accordance with relevant guidelines and regulations.

### 4.2. Population

A cohort of 62 newborns enrolled in the HYPOTOP trial was included in this study according to pre-established inclusion and exclusion criteria. The HYPOTOP trial is a randomized, controlled, multicenter, double-blinded clinical trial aiming to assess the efficacy of topiramate vs. placebo in patients with HIE undergoing TH. A detailed description of the HYPOTOP trial including the study design and sample-size estimation can be found elsewhere [31].

### 4.3. Magnetic Resonance Imaging

MRI was performed between Days 4 and 8 after birth. It was carried out using a 3T magnet system (Signa Excite^®^, General Electric Healthcare, IL, USA) and always included 3D Gradient Echo T1 weighted MR images, coronal and axial Fast Spin Echo T2 weighted MR images, diffusion weighted images (b0 and b1000 values), and susceptibility weighted imaging. In most cases, single voxel MR spectroscopy in thalamus, white, and grey matter was acquired using PROBE sequence, as well as diffusion tensor imaging (32 directions). The score used to differentiate mild from moderate and severe HIE was based on the MR score described by Barkovich [32]. Acute hypoxic ischemic insult in basal ganglia and thalami (BGT) was classified as mild if focal injury with normal posterior limb of the internal capsule (PLIC) was seen; as moderate if multifocal BGT pattern and equivocal or normal PLIC was identified; and as severe when diffuse BGT patterns and abnormal PLIC was detected. To evaluate white matter injury as well as cortical injury, we defined mild injury when only periventricular white matter was involved and normal cortex was preserved, moderate when subcortical white matter and focal areas of cortical highlighting were identified, and severe when widespread abnormalities with extensive cortical involvement were described. MRI results were interpreted by experienced, blinded radiologists. Based on the results of MRI, patients were classified as “normal” (*N* = 22) or “pathologic” (*N* = 33) neuronal outcomes following the criteria explained elsewhere [33]. Seven patients were excluded from further analysis as no MRI results were available. Patient characteristics of the remaining 55 infants are shown in Table 1.

### 4.4. Blood Sampling, Processing and Storing

A 0.5 mL sample of whole blood was extracted at birth (umbilical cord blood, when available, or extracted as early as possible before the initiation of TH; *N* = 36) and at 24 (*N* = 50), 48 (*N* = 50), and 72 h (*N* = 52) after the administration of the first dose of medication or placebo from a peripheral vein using heparinized syringes (1% sodium heparin). Blood samples were centrifuged immediately at 1800× *g* and 20 °C during 10 min. Plasma was collected and stored at −80 °C until analysis.

In total, 188 samples were collected from 62 newborns with moderate or severe HIE. Patients were recruited at 13 sites following a stringent study protocol and written standard operating procedures to limit the probability of chance correlations due to bias, systematic errors, or flaws in the experimental design.

### 4.5. Analytical Methods

Sample analysis was centralized and performed by two blinded analytical chemists in randomized order.

#### 4.5.1. Determination of Lactate and Pyruvate

The determination of lactate and pyruvate was carried out in plasma samples employing a two-step oximation–silylation procedure and a validated GC-MS method, employing a 6890 GC 5973N electron impact quadrupole MS system as described elsewhere [20].

#### 4.5.2. Untargeted Metabolomic Analysis

Plasma samples were thawed on ice and homogenized on a Vortex S0200 mixer (LabNet, Edison, NJ, USA) during 10 s at maximum speed. Seventy-five microliters of cold (4 °C) CH_3_OH were added to 25 µL of plasma and samples were centrifuged at 16,000 × *g* during 15 min at 4 °C. Eighty microliters of supernatant were collected and evaporated to dryness on a miVac centrifugal vacuum concentrator at room temperature and re-dissolved in 60 µL de H_2_O:CH_3_CN (0.1% *v/v* HCOOH) 95:5 containing phenylalanine-D_5_, caffeine-D_9_, leucine enkephalin, and reserpine, each at a concentration of 2 µM. Blank extracts were prepared by replacing whole blood with 500 µL of H_2_O. A quality control (QC) sample was prepared by mixing 5 μL of each plasma sample and a total of 10 QC sample aliquots were processed as described for plasma samples.

Chromatographic separations were performed on an Agilent 1290 Infinity UHPLC chromatograph using a UPLC ACQUITY BEH C18 column (2.1 mm × 100 mm, 1.7 μm, Waters, Wexford, Ireland) and a flow rate of 400 μL min^−1^. Autosampler and column temperatures were set to 4 and 40 °C, respectively, and an injection volume of 4 μL was used. Initial conditions of 98% of mobile phase A (H_2_O (0.1% *v/v* HCOOH)) were kept for 0.5 min, followed by a linear gradient from 2% to 20% of mobile phase B (CH_3_CN (0.1% *v/v* HCOOH)) in 3.5 min and from 20% to 95% B in 4 min. Conditions of 95% B were held for 1 min and a 0.25 min gradient was used to return to the initial conditions, which were held for 2.75 min.

Full-scan MS data between 100 and 1700 m/z with a scan frequency of 6 Hz (1274 transients/spectrum) were collected on an iFunnel quadrupole time-of-flight Agilent 6550 spectrometer operating in ESI^+^ and TOF MS mode. The electrospray ionization parameters were set as follows: gas T, 200 °C; drying gas, 14 L min^−1^; nebulizer, 37 psig; sheath gas T, 350 °C; sheath gas flow, 11 L min^−1^. For automatic MS spectra re-calibration during analysis, a mass reference standard was introduced into the source via a reference sprayer valve using 149.02332 (background contaminant), 121.050873 (purine), and 922.009798 (HP-0921) *m/z* as references.

System suitability was checked employing a standard mixture containing internal standards. The analytical system was conditioned by injecting the QC extract 9 times at the beginning of the batch. Data acquired during system conditioning were discarded from data analysis. In total, 188 plasma sample extracts were analyzed in random order in a single analytical batch. QC samples were analyzed every 5th sample and twice at the beginning and end of the batch for assessment and correction of instrumental performance [34]. Furthermore, automated UHPLC-ESI(+)-QqTOF (MS/MS) analysis of the QC sample was performed using two collision energies (20 and 40 V) to support metabolite annotation. The blank extract was injected a total of 4 times and used for data clean-up with the aim of identifying signals from other than biological origin.

#### 4.5.3. Statistics

Raw UHPLC-TOFMS data were converted into centroid mzXML format using ProteoWizard [35]. Metabolomics data have been deposited to the EMBL-EBI MetaboLights database with the identifier MTBLS1041 and the complete dataset can be accessed here [36]. A peak table was extracted using XCMS (version 3.4.2) [37] running in R (version 3.5). A total of 38014 features were initially detected after peak detection, integration, chromatographic deconvolution, and alignment. The CAMERA [38] package was used for identifying peak groups and annotation of isotopes and adducts.

The obtained peak table was imported into MATLAB R2017b (Mathworks Inc., Natick, MA, USA) for further data processing and analysis. To reduce intra-batch variation, the Quality Control-Support Vector Regression algorithm [39] and the LIBSVM library [40] were applied using an ε–range of 2.5 to 7.5, a γ-range of 1 to 10^5^. C was defined for each feature as the median value in QCs. Then, features detected in blanks (i.e., median peak area in QCs < 5 times the maximum value detected in blanks) and those with a RSD% in QC samples ≥20% were excluded. The final peak table contained 8122 features. Systematic identification of detected features was carried out by matching *m/z* values against the Human Metabolome Database (version 4.0) [41] with 5 ppm accuracy. To increase confidence in the identification, MS/MS fragmentation spectra were matched against reference spectra from HMDB and METLIN databases. Peak intensities of internal standards and QC samples were used for monitoring the instrumental response during data acquisition throughout the batch as described elsewhere [34,39].

MATLAB 2017b inbuilt functions as well as in-house written scripts (available from the authors upon reasonable request) and the PLS Toolbox 8.0 from Eigenvector Research Inc. (Wenatchee, WA, USA) were used for PCA, non-parametric Wilcoxon rank sum test, and *t*-test (*α* = 0.05, unequal variances). MetaboAnalyst (version 4.0) [42] was used for the construction of ROC curves, hierarchical clustering, and the generation of heat maps from auto scaled data employing Euclidean distance and Ward’s method. As an input for pathway analysis, *t*-test *p*-values (*α* = 0.05, unequal variances) and fold changes were computed for each metabolic feature comparing normal and pathologic outcomes for each sampling time point separately. Pathway analysis was carried out using MetaboAnalyst with the “MS peaks to pathways” tool (mass accuracy = 5 ppm; *mummichog* algorithm with *p*-value cutoff set to the top 10% of peaks) and the Kyoto Encyclopedia of Genes and Genomes (KEGG) pathway library (*Homo sapiens*). Pathways with *p*-values from Fisher’s exact *t*-test < 0.05 were considered as altered. Putative identifications based on *m/z* from pathway analysis were corroborated by manual inspection of raw data and MS/MS data when available (“level 2” identification as defined by The Metabolomics Standards Initiative [43]).

## Figures and Tables

**Table 1:** Clinical and demographic data of newborns.

Parameter	Normal (*N* = 22)	Pathologic (*N* = 33)	*p*-Value
Inborn	3 (14%)	8 (24%)	0.5
Maternal age (years)	34 (5)	33 (6)	0.4
Gestational age (weeks)	38.4 (37.3, 40.6)	39.0 (38.0, 40.6)	0.3
Gender (male, %)	13 (59%)	14 (42%)	0.3
Birth weight (g)	3265 (552)	3295 (697)	0.4
Length (cm)	51 (3)	51 (3)	0.4
Head circumference (cm)	34 (2)	34 (2)	0.3
Delivery mode (C-section)	9 (41%)	21 (64%)	0.2
Apgar score 1 min	2 (1, 4)	1 (1, 3)	0.6
Apgar score 5 min	4 (2, 5)	3 (1, 5)	0.3
Apgar score 10 min	5 (4, 6)	5 (4, 6)	1.0
Sarnat 2/Sarnat 3	19/3	19/14	0.05
pH UC	7.06 (0.27)	6.97 (0.17)	0.09
BE UC	−13 (10)	−16 (7)	0.2
pCO_2_ (mmHg) UC	71 (33)	59 (33)	0.2
HCO_3_ UC	15 (6)	13 (6)	0.2
Topiramate treatment (yes, %)	10 (45%)	17 (52%)	0.7
MR (days)	7 (5, 10)	7 (7, 10)	0.4

Note: Values are mean (SD), median (interquartile range), or *n* (%); *p*-values have been calculated employing the Student’s t-test for unequal variances (*α* = 0.05), the Wilcoxon rank sum test (*α* = 0.05) or the χ^2^-test (*α* = 0.05); UC, umbilical cord; BE, base excess; MR, magnetic resonance; inborn refers to infants born at the hospital where hypothermia treatment was carried out.

**Table 2:** Altered pathways in newborns suffering from HIE with pathologic MR outcomes.

Pathway	Total # of Metabolites	Hits (Total)	T0		T24		T48		T72	
			Hits (Sig)	Fisher’s *p*-Value	Hits (Sig)	Fisher’s *p*-Value	Hits (Sig)	Fisher’s *p*-Value	Hits (Sig)	Fisher’s *p*-Value
Alanine, aspartate, and glutamate metabolism	24	19	6	0.14	5	0.9	13	0.4	16	0.04
Arginine and proline metabolism	77	50	10	0.5	21	0.4	41	0.002	41	0.003
Caffeine metabolism	21	2	2	0.04	2	0.2	2	0.4	2	0.4
D-Glutamine and D-glutamate metabolism	11	7	1	0.8	3	0.6	7	0.03	7	0.04
Limonene and pinene degradation	59	7	2	0.4	1	1.0	4	0.7	7	0.04
Lysine biosynthesis	32	20	3	0.8	11	0.10	17	0.02	19	0.0013
Lysine degradation	47	32	7	0.4	17	0.07	21	0.4	28	0.002
Nitrogen metabolism	39	16	4	0.4	7	0.4	11	0.4	14	0.03
Phenylalanine metabolism	45	25	5	0.5	15	0.02	24	0.00009	21	0.02
Seleno amino acid metabolism	22	2	2	0.04	1	0.6	2	0.4	2	0.4
Steroid hormone biosynthesis	99	29	11	0.01	23	0.000009	25	0.004	25	0.006

Note: Results with a Fisher’s *p*-value < 0.05 are gray-shaded.

## Supplementary Materials

The following are available online at https://www.mdpi.com/2218-1989/10/3/109/s1, Table S1: Annotation of altered metabolites. Note: Blue stands for detected, but not altered; red stands for altered; IDs of annotated metabolites marked with a star have been verified with MS/MS data.

## Appendix A

Members of the HYPOTOP Study Group: Isabel Izquierdo, Ana Gimeno, María Gormaz, Raquel Escrig, María Cernada, Marta Aguar, Ester Torres (Division of Neonatology, University and Polytechnic Hospital La Fe, Valencia, Spain); Isabel Benavente-Fernández (Division of Neonatology, University Hospital Puerta del Mar, Cádiz, Spain); Eva Valverde, Malaika Cordeiro (Division of Neonatology, University Hospital La Paz, Madrid, Spain); Dorotea Blanco (Division of Neonatology, University Hospital Gregorio Marañón, Madrid, Spain); Hector Boix (Department of Neonatology, University Hospital Vall d’Hebrón, Barcelona, Spain); Fernando Cabañas (Division of Neonatology, University Hospital Quirónsalud Madrid, Madrid, Spain); Mercedes Chaffanel (Division of Neonatology, Regional University Hospital Málaga, Málaga, Spain); Belén Fernández-Colomer (Division of Neonatology, Central University Hospital of Asturias, Oviedo, Spain); Jose Ramón Fernández-Lorenzo (Division of Neonatology, University Hospital Complex of Vigo, Vigo, Spain); Begoña Loureiro (Division of Neonatology, University Hospital Cruces, Bilbao, Spain); Maria Teresa Moral-Pumarega (Division of Neonatology, University Hospital 12 de Octubre, Madrid, Spain); Antonio Pavón (Division of Neonatology, University Hospital Virgen del Rocío, Sevilla, Spain); and Inés Tofé (Division of Neonatology, University Hospital Reina Sofía, Córdoba, Spain).

## References

[1] Kurinczuk J.J., Barralet J.H., Redshaw M., Brocklehurst P. (2005). Report to the Patient Safety Research Programme (Policy Research Programme of the Department Of Health). https://www.npeu.ox.ac.uk/downloads/files/reports/coNsEnsus-Monitoring-NE-Report.pdf.

[2] Douglas-Escobar M., Weiss M.D. (2015). Hypoxic-ischemic encephalopathy: A review for the clinician. JAMA Pediatr..

[3] Lee A.C.C., Kozuki N., Blencowe H., Vos T., Bahalim A., Darmstadt G.L., Niermeyer S., Ellis M., Robertson N.J., Cousens S. (2013). Intrapartum-related neonatal encephalopathy incidence and impairment at regional and global levels for 2010 with trends from 1990. Pediatr. Res..

[4] Wassink G., Davidson J.O., Dhillon S.K., Zhou K., Bennet L., Thoresen M., Gunn A.J. (2019). Therapeutic Hypothermia in Neonatal Hypoxic-Ischemic Encephalopathy. Curr. Neurol. Neurosci. Rep..

[5] Sarnat H.B., Sarnat M.S. (1976). Neonatal encephalopathy following fetal distress. A clinical and electroencephalographic study. Arch. Neurol..

[6] Merchant N., Azzopardi D. (2015). Early predictors of outcome in infants treated with hypothermia for hypoxic-ischaemic encephalopathy. Dev. Med. Child Neurol..

[7] Groenendaal F., de Vries L.S. (2017). Fifty years of brain imaging in neonatal encephalopathy following perinatal asphyxia. Pediatr. Res..

[8] Shibasaki J., Aida N., Morisaki N., Tomiyasu M., Nishi Y., Toyoshima K. (2018). Changes in Brain Metabolite Concentrations after Neonatal Hypoxic-ischemic Encephalopathy. Radiology.

[9] Lee W.L.A., Michael-Titus A.T., Shah D.K. (2017). Hypoxic-Ischaemic Encephalopathy and the Blood-Brain Barrier in Neonates. Dev. Neurosci..

[10] Bennet L., Booth L., Gunn A.J. (2010). Potential biomarkers for hypoxic-ischemic encephalopathy. Semin. Fetal Neonatal Med..

[11] Sánchez-Illana Á., Piñeiro-Ramos J.D., Kuligowski J. (2020). Small molecule biomarkers for neonatal hypoxic ischemic encephalopathy. Semin. Fetal Neonatal Med..

[12] Wu T.-W., Tamrazi B., Hsu K.-H., Ho E., Reitman A.J., Borzage M., Blüml S., Wisnowski J.L. (2018). Cerebral Lactate Concentration in Neonatal Hypoxic-Ischemic Encephalopathy: In Relation to Time, Characteristic of Injury, and Serum Lactate Concentration. Front. Neurol..

[13] Vannucci R.C., Brucklacher R.M., Vannucci S.J. (2005). Glycolysis and Perinatal Hypoxic-Ischemic Brain Damage. Dev. Neurosci..

[14] Efstathiou N., Theodoridis G., Sarafidis K. (2017). Understanding neonatal hypoxic-ischemic encephalopathy with metabolomics. Hippokratia.

[15] Reinke S.N., Walsh B.H., Boylan G.B., Sykes B.D., Kenny L.C., Murray D.M., Broadhurst D.I. (2013). 1H NMR derived metabolomic profile of neonatal asphyxia in umbilical cord serum: Implications for hypoxic ischemic encephalopathy. J. Proteome Res..

[16] Ahearne C.E., Denihan N.M., Walsh B.H., Reinke S.N., Kenny L.C., Boylan G.B., Broadhurst D.I., Murray D.M. (2016). Early Cord Metabolite Index and Outcome in Perinatal Asphyxia and Hypoxic-Ischaemic Encephalopathy. Neonatology.

[17] Denihan N.M., Kirwan J.A., Walsh B.H., Dunn W.B., Broadhurst D.I., Boylan G.B., Murray D.M. (2017). Untargeted metabolomic analysis and pathway discovery in perinatal asphyxia and hypoxic-ischaemic encephalopathy. J. Cereb. Blood Flow Metab..

[18] Sarafidis K., Efstathiou N., Begou O., Soubasi V., Agakidou E., Gika E., Theodoridis G., Drossou V. (2017). Urine metabolomic profile in neonates with hypoxic-ischemic encephalopa-thy. Hippokratia.

[19] Yum S.K., Moon C.-J., Youn Y.-A., Sung I.K. (2017). Changes in lactate dehydrogenase are associated with central gray matter lesions in newborns with hypoxic-ischemic encephalopathy. J. Matern. Fetal Neonatal Med..

[20] Sánchez-Illana Á., Núñez-Ramiro A., Cernada M., Parra-Llorca A., Valverde E., Blanco D., Moral-Pumarega M.T., Cabañas F., Boix H., Pavon A. (2017). Evolution of Energy Related Metabolites in Plasma from Newborns with Hypoxic-Ischemic Encephalopathy during Hypothermia Treatment. Sci. Rep..

[21] Chiang M.-C., Lien R., Chu S.-M., Yang P.-H., Lin J.-J., Hsu J.-F., Fu R.-H., Lin K.-L. (2016). Serum Lactate, Brain Magnetic Resonance Imaging and Outcome of Neonatal Hypoxic Ischemic Encephalopathy after Therapeutic Hypothermia. Pediatr. Neonatol..

[22] Shah S., Tracy M., Smyth J. (2004). Postnatal lactate as an early predictor of short-term outcome after intrapartum asphyxia. J. Perinatol..

[23] Murray D.M., Boylan G.B., Fitzgerald A.P., Ryan C.A., Murphy B.P., Connolly S. (2008). Persistent lactic acidosis in neonatal hypoxic-ischaemic encephalopathy correlates with EEG grade and electrographic seizure burden. Arch. Dis. Child. Fetal Neonatal Ed..

[24] Beckstrom A.C., Tanya P., Humston E.M., Snyder L.R., Synovec R.E., Juul S.E. (2012). The perinatal transition of the circulating metabolome in a nonhuman primate. Pediatr. Res..

[25] Kuligowski J., Solberg R., Sánchez-Illana Á., Pankratov L., Parra-Llorca A., Quintás G., Saugstad O.D., Vento M. (2017). Plasma metabolite score correlates with Hypoxia time in a newly born piglet model for asphyxia. Redox Biol..

[26] Anandi V.S., Shaila B. (2017). Evaluation of factors associated with elevated newborn 17-hydroxyprogesterone levels. J. Pediatr. Endocrinol. Metab..

[27] Concepcion K.R., Zhang L. (2018). Corticosteroids and perinatal hypoxic-ischemic brain injury. Drug Discov. Today.

[28] Dong S., Zhang Q., Kong D., Zhou C., Zhou J., Han J., Zhou Y., Jin G., Hua X., Wang J. (2018). Gender difference in the effect of progesterone on neonatal hypoxic/ischemic brain injury in mouse. Exp. Neurol..

[29] Walsh B.H., Broadhurst D.I., Mandal R., Wishart D.S., Boylan G.B., Kenny L.C., Murray D.M. (2012). The Metabolomic Profile of Umbilical Cord Blood in Neonatal Hypoxic Ischaemic Encephalopathy. PLoS ONE.

[30] Kariholu U., Montaldo P., Markati T., Lally P.J., Pryce R., Teiserskas J., Liow N., Oliveira V., Soe A., Shankaran S. (2020). Therapeutic hypothermia for mild neonatal encephalopathy: A systematic review and meta-analysis. Arch. Dis. Child. Fetal Neonatal Ed..

[31] Nuñez-Ramiro A., Benavente-Fernández I., Valverde E., Cordeiro M., Blanco D., Boix H., Cabañas F., Chaffanel M., Fernández-Colomer B., Fernández-Lorenzo J.R. (2019). Topiramate plus Cooling for Hypoxic-Ischemic Encephalopathy: A Randomized, Controlled, Multicenter, Double-Blinded Trial. Neonatology.

[32] Barkovich A.J., Hajnal B.L., Vigneron D., Sola A., Partridge J.C., Allen F., Ferriero D.M. (1998). Prediction of neuromotor outcome in perinatal asphyxia: Evaluation of MR scoring systems. AJNR Am. J. Neuroradiol..

[33] Rutherford M., Ramenghi L.A., Edwards A.D., Brocklehurst P., Halliday H., Levene M., Strohm B., Thoresen M., Whitelaw A., Azzopardi D. (2010). Assessment of brain tissue injury after moderate hypothermia in neonates with hypoxic-ischaemic encephalopathy: A nested substudy of a randomised controlled trial. Lancet Neurol..

[34] Broadhurst D., Goodacre R., Reinke S.N., Kuligowski J., Wilson I.D., Lewis M.R., Dunn W.B. (2018). Guidelines and considerations for the use of system suitability and quality control samples in mass spectrometry assays applied in untargeted clinical metabolomic studies. Metabolomics.

[35] ProteoWizard. http://proteowizard.sourceforge.net.

[36] MetaboLights. https://www.ebi.ac.uk/metabolights/MTBLS1041.

[37] LC-MS and GC-MS Data Analysis. https://bioconductor.org/packages/release/bioc/html/xcms.html.

[38] Kuhl C., Tautenhahn R., Böttcher C., Larson T.R., Neumann S. (2012). CAMERA: An Integrated Strategy for Compound Spectra Extraction and Annotation of Liquid Chromatography/Mass Spectrometry Data Sets. Anal. Chem..

[39] Kuligowski J., Sánchez-Illana Á., Sanjuán-Herráez D., Vento M., Quintás G. (2015). Intra-batch effect correction in liquid chromatography-mass spectrometry using quality control samples and support vector regression (QC-SVRC). Analyst.

[40] Chang C.-C., Lin C.-J. (2011). LIBSVM: A Library for Support Vector Machines. ACM Trans. Intell. Syst. Technol..

[41] HMDB Version 4.0. http://www.hmdb.ca.

[42] Chong J., Soufan O., Li C., Caraus I., Li S., Bourque G., Wishart D.S., Xia J. (2018). MetaboAnalyst 4.0: Towards more transparent and integrative metabolomics analysis. Nucleic Acids Res..

[43] Sumner L.W., Amberg A., Barrett D., Beale M.H., Beger R., Daykin C.A., Fan T.W.-M., Fiehn O., Goodacre R., Griffin J.L. (2007). Proposed minimum reporting standards for chemical analysis Chemical Analysis Working Group (CAWG) Metabolomics Standards Initiative (MSI). Metabolomics.

