# Supplementary material for: Metabolic Phenotypes of Hypoxic-Ischemic Encephalopathy with Normal vs. Pathologic Magnetic Resonance Imaging Outcomes
Source: Metabolites. 2020 Mar 14;10(3):109. doi: 10.3390/metabo10030109 (PMC7143850; doi:10.3390/metabo10030109)
Supplement: Supplementary file 1 [file metabolites-10-00109-s001.pdf]

Supplementary Material to:

Metabolic Phenotypes of Hypoxic-Ischemic Encephalopathy

José David Piñeiro-Ramos[1], MSc; Antonio Núñez-Ramiro[2], MD; Roberto Llorens-Salvador[3], MD; Anna Parra-Llorca[1], MD; Ángel Sánchez-Illana[1], MSc; Guillermo Quintás[4,5], PhD; Nuria Boronat-Gonzalez[2], PhD; Juan Martínez-Rodilla[1], MSc; Julia Kuligowski[1]*, PhD; Máximo Vento[1,2**], PhD; and the Hypotop Study Group

[1]Neonatal Research Group, Health Research Institute Hospital La Fe, Avenida Fernando Abril Martorell 106, 46026 Valencia, Spain

[2]Division of Neonatology, University & Polytechnic Hospital La Fe, Avenida Fernando Abril Martorell 106, 46026 Valencia, Spain

[3]Division of Radiology and Imaging, University & Polytechnic Hospital La Fe, Avenida Fernando Abril Martorell 106, 46026 Valencia, Spain

[4]Human & Environmental Health & Safety (HEHS), Leitat Technological Center, Avenida Fernando Abril Martorell 106, 46026 Valencia, Spain

[5]Unidad Analítica, Health Research Institute La Fe, Avenida Fernando Abril Martorell 106, 46026 Valencia, Spain

*Corresponding Author: Julia Kuligowski PhD; Address: Avenida Fernando Abril Martorell 106, 46026 Valencia, Spain; Phone: +34/961246661; e-mail: julia.kuligowski@uv.es

**Senior Author: Maximo Vento MD PhD; Address: Avenida Fernando Abril Martorell 106, 46026 Valencia, Spain; Phone: +34/961245688; e-mail: maximo.vento@uv.es

Supplementary Table 1. Annotation of altered metabolites. Note: blue stands for detected, but not altered; red stands for altered; IDs of annotated metabolites marked with a star have been verified with MS/MS data.

Pathway	KEGG	Name	Mol Form	T0	T24	T48	T72	Adduct	m/z
**Steroid hormone biosynthesis**	C00735	Cortisol	C21H30O5	blue	red	red	red	[M+H]+	363.2166
	C01780	Aldosterone	C21H28O5	blue	red	red	red	[M+H]+	361.2010
	C01124	18-Hydroxycorticosterone	C21H30O5	blue	red	red	red	[M+H]+	363.2166
	C00187	Cholesterol	C27H46O	blue	blue	red	blue	[M+HCOONa]+	455.3496
	C01953	Pregnenolone	C21H32O2	red	red	red	red	[M+H]+	317.2475
	C01227	Dehydroepiandrosterone	C19H28O2	red	red	red	red	[M+Na]+	311.1982
	C04295	Androstenediol	C19H30O2	red	red	red	red	[M+Na]+	313.2138
	C03205	11-Deoxycorticosterone	C21H30O3	red	red	red	red	[M+H]+	331.2268
	C02140	Corticosterone	C21H30O4	red	red	red	red	[M+H]+	347.2217
	C00410	Progesterone	C21H30O2	blue	red	red	red	[M+H]+	315.2319
	C03681	5alpha-Pregnane-3,20-dione	C21H32O2	red	red	red	red	[M+H]+	317.2475
	C01176	17alpha-Hydroxyprogesterone	C21H30O3	red	red	red	red	[M+H]+	331.2268
	C00762	Cortisone	C21H28O5	blue	red	red	red	[M+H]+	361.2010
	C00535	Testosterone	C19H28O2	red	red	red	red	[M+Na]+	311.1982
	C00280	Androstenedione*	C19H26O2	blue	blue	blue	red	[M+H]+	287.2006
	C03772	5beta-Androstane-3,17-dione	C19H28O2	red	red	red	red	[M+Na]+	311.1982
	C04373	Etiocholanolone	C19H30O2	red	red	red	red	[M+Na]+	313.2138
	C00674	5alpha-Androstane-3,17-dione	C19H28O2	red	red	red	red	[M+Na]+	311.1982
	C00523	Androsterone	C19H30O2	red	red	red	red	[M+Na]+	313.2138
	C00468	Estrone	C18H22O2	blue	red	red	red	[M+H]+	271.1693
	C03917	Dihydrotestosterone	C19H30O2	red	red	red	red	[M+Na]+	313.2138
	C02538	Estrone 3-sulfate	C18H22O5S	blue	blue	red	red	[M-H2O+H]+	333.1155
	C03747	11alpha-Hydroxyprogesterone	C21H30O3	red	red	red	red	[M+H]+	331.2268
	C04042	20alpha-Hydroxy-4-pregnen-3-one	C21H32O2	red	red	red	red	[M+H]+	317.2475
	C03852	Androstan-3alpha,17beta-diol	C19H32O2	red	red	red	red	[M+Na]+	315.2295
	C03935	6beta-Hydroxy-17beta-estradiol	C18H24O3	blue	red	red	red	[M+K]+	327.1357
**Caffeine**	C01762	Xanthosine	C10H12N4O6	red	blue	blue	blue	[M+H]+	285.0830
**metabolism**	C00385	Xanthine*	C5H4N4O2	red	blue	blue	blue	[M+H]+	153.0407
**Selenoamino**	C00979	O-Acetyl-L-serine	C5H9NO4	red	blue	blue	blue	[M+H]+	148.0604
**acid metabolism**	C00041	L-Alanine	C3H7NO2	red	blue	blue	blue	[M+H]+	90.0550

Phenylalanine metabolism	C03589	4-Hydroxy-2-oxopentanoate	C5H8O4					[M+H]+	133.0495
	C00079	L-Phenylalanine	C9H11NO2					[M+H]+	166.0863
	C00601	Phenylacetaldehyde	C8H8O					[M-H2O+H]+	103.0542
	C00596	2-Hydroxy-2,4-pentadienoate	C5H6O3					[M+H2O+H]+	133.0495
	C04044	3-(2,3-Dihydroxyphenyl)propanoate	C9H10O4					[M+H]+	183.0652
	C00166	Phenylpyruvate	C9H8O3					[M+H]+	165.0546
	C02265	D-Phenylalanine	C9H11NO2					[M+H]+	166.0863
	C02505	2-Phenylacetamide	C8H9NO					[M+H]+	136.0757
	C00423	trans-Cinnamate	C9H8O2					[M+H]+	149.0597
	C01198	3-(2-Hydroxyphenyl)propanoate	C9H10O3					[M+H]+	167.0703
	C00180	Benzoate	C7H6O2					[M+H]+	123.0441
	C01586	Hippurate	C9H9NO3					[M+H]+	180.0655
	C01722	trans-2-Hydroxycinnamate	C9H8O3					[M+H]+	165.0546
	C00811	4-Coumarate	C9H8O3					[M+H]+	165.0546
	C02137	alpha-Oxo-benzeneacetic acid	C8H6O3					[M+H]+	151.0390
	C04148	Phenylacetylglutamine	C13H16N2O4					[M+H]+	265.1183
	C00042	Succinate	C4H6O4					[M+H]+	119.0339
	C00122	Fumarate	C4H4O4					[M-CO2+H]+	73.0284
	C02763	2-Hydroxy-3-phenylpropenoate	C9H8O3					[M+H]+	165.0546
	C03519	N-Acetyl-L-phenylalanine	C11H13NO3					[M+K]+	246.0527
	C00156	4-Hydroxybenzoate	C7H6O3					[M+H]+	139.0390
	C00642	4-Hydroxyphenylacetate	C8H8O3					[M+H]+	153.0546
	C00805	Salicylate	C7H6O3					[M+H]+	139.0390
	C00082	L-Tyrosine*	C9H11NO3					[M+H]+	182.0812
Arginine and proline metabolism	C01165	L-Glutamate 5-semialdehyde	C5H9NO3					[M+H]+	132.0655
	C04281	L-1-Pyrroline-3-hydroxy-5-carboxylate	C5H7NO3					[M+H]+	130.0499
	C00064	L-Glutamine	C5H10N2O3					[M+H]+	147.0764
	C00077	L-Ornithine	C5H12N2O2					[M+H]+	133.0972
	C00049	L-Aspartate	C4H7NO4					[M+H2O+H]+	152.0548
	C03406	L-Argininosuccinate	C10H18N4O6					[M+H]+	291.1299
	C00025	L-Glutamate	C5H9NO4					[M+H]+	148.0604
	C00624	N-Acetyl-L-glutamate	C7H11NO5					[M+H]+	190.0710
	C04133	N-Acetyl-L-glutamate 5-phosphate	C7H12NO8P					[M-CO+H]+	242.0432
	C01250	N-Acetyl-L-glutamate 5-semialdehyde	C7H11NO4					[M+H]+	174.0761

Arginine and proline metabolism	C00437	N-Acetylornithine	C7H14N2O3	[M+H]+	175.1077
	C00148	L-Proline	C5H9NO2	[M+H]+	116.0706
	C01877	4-Oxoproline	C5H7NO3	[M+H]+	130.0499
	C00763	D-Proline	C5H9NO2	[M+H]+	116.0706
	C03564	1-Pyrroline-2-carboxylate	C5H7NO2	[M+H]+	114.0550
	C01157	Hydroxyproline	C5H9NO3	[M+H]+	132.0655
	C01682	Nopaline	C11H20N4O6	[M-NH3+H]+	288.1185
	C00300	Creatine	C4H9N3O2	[M+H]+	132.0768
	C01043	N-Carbamoylsarcosine	C4H8N2O3	[M+NaCl]+	191.0179
	C00791	Creatinine	C4H7N3O	[M+H]+	114.0662
	C03771	5-Guanidino-2-oxopentanoate	C6H11N3O3	[M+H]+	174.0874
	C02647	4-Guanidinobutanal	C5H11N3O	[M+Na]+	152.0787
	C01035	4-Guanidinobutanoate	C5H11N3O2	[M+H]+	146.0924
	C00334	4-Aminobutanoate	C4H9NO2	[M+H]+	104.0706
	C03078	4-Guanidinobutanamide	C5H12N4O	[M+Na]+	167.0896
	C00436	N-Carbamoylputrescine	C5H13N3O	[M+NaCl]+	190.0722
	C03296	N2-Succinyl-L-arginine	C10H18N4O5	[M+NaCl]+	333.0910
	C03415	N2-Succinyl-L-ornithine	C9H16N2O5	[M+H]+	233.1132
	C03912	(S)-1-Pyrroline-5-carboxylate	C5H7NO2	[M+H]+	114.0550
	C00555	4-Aminobutyraldehyde	C4H9NO	[M+H]+	88.0757
	C01137	S-Adenosylmethioninamine	C14H23N6O3S	[M+H]+	356.1602
	C00019	S-Adenosyl-L-methionine	C15H22N6O5S	[M+2H]+	200.0743
	C02714	N-Acetylputrescine	C6H14N2O	[M+H]+	131.1179
	C02946	4-Acetamidobutanoate	C6H11NO3	[M+H]+	146.0812
	C03440	cis-4-Hydroxy-D-proline	C5H9NO3	[M+H]+	132.0653
	C04282	1-Pyrroline-4-hydroxy-2-carboxylate	C5H7NO3	[M+H]+	130.0499
	C00122	Fumarate	C4H4O4	[M-CO2+H]+	73.0286
	C01110	5-Amino-2-oxopentanoic acid	C5H9NO3	[M+H]+	132.0655
	C00431	5-Aminopentanoate	C5H11NO2	[M+H]+	118.0863
	C04137	D-Octopine	C9H18N4O4	[M+H]+	247.1401
	C00213	Sarcosine	C3H7NO2	[M+H]+	90.0550
	C00884	Homocarnosine	C10H16N4O3	[M+H]+	241.1295
	C03166	Phosphoguanidinoacetate	C3H8N3O5P	[M-CO+H]+	170.0330

Lysine biosynthesis	C00666	LL-2,6-Diaminoheptanedioate	C7H14N2O4					[M+H]+	191.1026
	C00956	L-2-Aminoadipate	C6H11NO4					[M+H]+	162.0761
	C00049	L-Aspartate	C4H7NO4					[M+H2O+H]+	152.0548
	C03082	4-Phospho-L-aspartate	C4H8NO7P					[M-HCOOK+H]+	130.0499
	C00263	L-Homoserine	C4H9NO3					[M+H]+	120.0655
	C00441	L-Aspartate 4-semialdehyde	C4H7NO3					[M+H2O+H]+	136.0618
	C03972	2,3,4,5-Tetrahydrodipicolinate	C7H9NO4					[M-H2O+H]+	190.0722
	C03871	L-2-Amino-6-oxoheptanedioate	C7H11NO5					[M+H]+	190.0723
	C04421	N-Succinyl-LL-2,6-diaminoheptanedioate	C11H18N2O7					[M+H2O+H]+	309.1265
	C04390	N6-Acetyl-LL-2,6-diaminoheptanedioate	C9H16N2O5					[M+H]+	233.1132
	C00680	meso-2,6-Diaminoheptanedioate	C7H14N2O4					[M+H]+	191.1029
	C00449	N6-(L-1,3-Dicarboxypropyl)-L-lysine	C11H20N2O6					[M+H]+	277.1394
	C01251	Homocitrate	C7H10O7					[M+H]+	207.0516
	C00026	2-Oxoglutarate	C5H6O5					[M+HCOONa]+	215.0166
	C00322	2-Oxoadipate	C6H8O5					[M+H]+	161.0460
	C03340	Dihydrodipicolinate	C7H7NO4					M-CO2+H[1+]	126.0549
	C04462	N-Succinyl-2-L-amino-6-oxoheptanedioate	C11H15NO8					[M+H]+	290.0854
	C04076	L-2-Aminoadipate 6-semialdehyde	C6H11NO3					[M+H]+	146.0812
	C04002	cis-Homoaconitate	C7H8O6					[M+Na]+	211.0209
D-Glutamine and D-glutamate metabolism	C00819	D-Glutamine	C5H10N2O3					[M+H]+	147.0764
	C00217	D-Glutamate	C5H9NO4					[M+H]+	148.0604
	C01212	UDP-N-acetylmuramoyl-L-alanine	C23H36N4O20P2					M(S34)+H[1+]	753.1441
	C00025	L-Glutamate	C5H9NO4					[M+H]+	148.0604
	C00064	L-Glutamine	C5H10N2O3					[M+H]+	147.0764
	C02237	5-Oxo-D-proline	C5H7NO3					[M+H]+	130.0499
	C00026	2-Oxoglutarate	C5H6O5					[M+HCOONa]+	215.0166
Lysine degradation	C00739	D-Lysine	C6H14N2O2					[M+H]+	147.1128
	C00047	L-Lysine	C6H14N2O2					[M+H]+	147.1128
	C04092	Delta1-Piperideine-2-carboxylate	C6H9NO2					[M+H]+	128.0706
	C00449	Saccharopine	C11H20N2O6					[M+H]+	277.1403
	C01149	4-Trimethylammoniobutanal	C7H16NO					[M+NaCl]+	189.0897
	C03793	N6,N6,N6-Trimethyl-L-lysine	C9H20N2O2					[M+H]+	189.1598
	C01144	(S)-3-Hydroxybutanoyl-CoA	C25H42N7O18P3S					[M+NaCl]+	912.1245
	C00408	Pipecolinic acid	C6H11NO2					[M+H]+	130.0863

Lysine degradation	C02727	N6-Acetyl-L-lysine	C8H16N2O3	[M+H]+	189.1234
	C04076	L-2-Aminoadipate 6-semialdehyde	C6H11NO3	[M+H]+	146.0812
	C00956	L-2-Aminoadipate	C6H11NO4	[M+H]+	162.0761
	C00322	2-Oxoadipate	C6H8O5	[M+H]+	161.0444
	C00527	Glutaryl-CoA	C26H42N7O19P3S	M(C13)+2H[2+]	442.0844
	C00489	Glutaric acid	C5H8O4	[M+H]+	133.0495
	C03273	5-Oxopentanoate	C5H8O3	M+[HCOONa]+	185.0421
	C00990	5-Aminopentanamide	C5H12N2O	[M-NH3+H]+	100.0758
	C03087	5-Acetamidopentanoate	C7H13NO3	[M+H]+	160.0968
	C01142	L-beta-Lysine	C6H14N2O2	[M+H]+	147.1128
	C01186	(3S,5S)-3,5-Diaminohexanoate	C6H14N2O2	[M+H]+	147.1128
	C03955	N6-Acetyl-N6-hydroxy-L-lysine	C8H16N2O4	[M+H]+	205.1183
	C01028	N6-Hydroxy-L-lysine	C6H14N2O3	[M+H]+	163.1077
	C04020	D-Lysopine	C9H18N2O4	[M+H]+	219.1339
	C03239	6-Amino-2-oxohexanoate	C6H11NO3	[M+H]+	146.0812
	C00487	Carnitine	C7H16NO3	[M+NaCl]+	221.0796
	C03366	5-Phosphooxy-L-lysine	C6H15N2O6P	[M+H2O+H]+	261.0872
	C00450	(S)-2,3,4,5-Tetrahydropyridine-2-carboxylate	C6H9NO2	[M+H]+	128.0706
	C00431	5-Aminopentanoate	C5H11NO2	[M+H]+	118.0863
	C03656	(S)-5-Amino-3-oxohexanoic acid	C6H11NO3	[M+H]+	146.0812
Nitrogen metabolism	C00079	L-Phenylalanine	C9H11NO2	[M+H]+	166.0863
	C00082	L-Tyrosine*	C9H11NO3	[M+H]+	182.0812
	C00078	L-Tryptophan*	C11H12N2O2	[M+H]+	205.0972
	C03618	L-threo-3-Methylaspartate	C5H9NO4	[M+H]+	148.0604
	C00049	L-Aspartate	C4H7NO4	[M+H2O+H]+	152.0569
	C00152	L-Asparagine	C4H8N2O3	[M+NaCl]+	191.0179
	C00025	L-Glutamate	C5H9NO4	[M+H]+	148.0604
	C00064	L-Glutamine	C5H10N2O3	[M+H]+	147.0764
	C00108	Anthranilic acid	C7H7NO2	[M+H]+	138.0550
	C02291	L-Cystathionine	C7H14N2O4S	[M+Na]+	245.0589
	C00155	L-Homocysteine	C4H9NO2S	[M-NH3+H]+	119.0162
	C00542	Cystathionine	C7H14N2O4S	[M+Na]+	245.0589
	C00135	L-Histidine	C6H9N3O2	[M+H]+	156.0768
	C00020	Adenylic acid	C10H14N5O7P	[M+H]+	348.0704

**Limonene and pinene degradation**	C02759	alpha-Pinene-oxide	C10H16O					[M+K]+	191.0817
	C04435	(Z)-2-Methyl-5-isopropylhexa-2,5-dienal	C10H16O					[M+K]+	191.0817
	C02676	Perillyl aldehyde	C10H14O					[M+Na]+	151.1119
	C01767	(-)-Carvone	C10H14O					[M+Na]+	151.1119
	C00964	(-)-trans-Carveol	C10H16O					[M+K]+	191.0817
	C02452	Perillyl alcohol	C10H16O					[M+K]+	191.0817
	C00521	(-)-Limonene	C10H16					[M+K]+	175.0869
**Alanine, aspartate and glutamate metabolism**	C01042	N-Acetyl-L-aspartate	C6H9NO5					[M-HCOOH+H]+	130.0498
	C02362	2-Oxosuccinamate	C4H5NO4					[M+K]+	169.9862
	C00049	L-Aspartate	C4H7NO4					[M+H2O+H]+	152.0569
	C00152	L-Asparagine	C4H8N2O3					[M+H]+	133.0608
	C00402	D-Aspartate	C4H7NO4					[M+H2O+H]+	152.0569
	C03406	L-Argininosuccinate	C10H18N4O6					[M+H]+	291.1299
	C00041	L-Alanine	C3H7NO2					[M+H]+	90.0550
	C00026	2-Oxoglutarate	C5H6O5					[M+HCOONa]+	215.0166
	C00064	L-Glutamine	C5H10N2O3					[M+H]+	147.0764
	C00025	L-Glutamate	C5H9NO4					[M+H]+	148.0604
	C00334	gamma-Aminobutyric acid	C4H9NO2					[M+H]+	104.0706
	C00940	2-Oxoglutaramate	C5H7NO4					[M+K]+	184.0002
	C03912	L-1-Pyrroline-5-carboxylate	C5H7NO2					[M+H]+	114.0550
	C00042	Succinic acid	C4H6O4					[M+H]+	119.0339
	C00352	D-Glucosamine 6-phosphate	C6H14NO8P					[M+H]+	260.0530
	C03090	5-Phosphoribosylamine	C5H12NO7P					[M-CO+H]+	202.0476
